# Segmentation of Regions of Interest Using Active Contours with SPF Function

**DOI:** 10.1155/2015/710326

**Published:** 2015-05-18

**Authors:** Farhan Akram, Jeong Heon Kim, Chan-Gun Lee, Kwang Nam Choi

**Affiliations:** ^1^Department of Computer Engineering and Mathematics, Rovira i Virgili University, 43007 Tarragona, Spain; ^2^Department of Computer Science & Engineering, Chung-Ang University, Seoul 156-756, Republic of Korea; ^3^Korea Institute of Science & Technology Information, Daejeon 305-806, Republic of Korea

## Abstract

Segmentation of regions of interest is a well-known problem in image segmentation. This paper presents a region-based image segmentation technique using active contours with signed pressure force (SPF) function. The proposed algorithm contemporaneously traces high intensity or dense regions in an image by evolving the contour inwards. In medical image modalities these high intensity or dense regions refer to tumor, masses, or dense tissues. The proposed method partitions an image into an arbitrary number of subregions and tracks down salient regions step by step. It is implemented by enforcing a new region-based SPF function in a traditional edge-based level set model. It partitions an image into subregions and then discards outer subregion and partitions inner region into two more subregions; this continues iteratively until a stopping condition is fulfilled. A Gaussian kernel is used to regularize the level set function, which not only regularizes it but also removes the need of computationally expensive reinitialization. The proposed segmentation algorithm has been applied to different images in order to demonstrate the accuracy, effectiveness, and robustness of the algorithm.

## 1. Introduction

The advanced imaging technologies have improved significantly the quality of medical care for patients. These technologies allowed a radiologist to make increasingly accurate diagnoses of suspicious regions like tumors, polyps, and blood rupture areas and helped physicians to render precise and measured modes of treatment [1]. It is usually a difficult task to identify significant information in a medical image because of intensity inhomogeneity and blurred object boundaries. Expert radiologists are needed to analyze the region of interest (ROI) in a particular image modality, which is costly and time consuming job. There we need an automated ROI system, which can prompt a particular region of interest and can help untrained personnel, saving both time and money. A region of interest analysis is a fundamental step in a computer-aided diagnosis (CAD) system for medical imaging, which helps early detection of cancer [2]. It prompts suspicious regions in different medical images such as magnetic resonance imaging (MRI), X-ray imaging, and computerized tomography (CT) imaging. The necessity of thoroughly examining a large number of image modalities in order to detect small number of cancers can cause high false positive, which can lead to unnecessary biopsies. Moreover, some of the salient regions can be missed due to radiologist's tiredness or distraction [3, 4]. A system which segments regions of interest can help radiologists by tapering their search only to the desired objects in an image. Use of this type of CAD system can increase the efficiency of correct detection of tumors and decrease number of false positives found by the radiologists [5, 6]. Numerous approaches have been developed to solve this tedious but necessary problem of ROI identification and segmentation in the postprocessing of cancer research and treatment. In late 1980s, Kass et al. introduced an active contour method, which is one of well-known techniques used to segment ROI in the computer vision and image processing applications [7]. In this method, a curve is evolved under a force by minimizing the energy until it stops at the object boundary. The energy functional is normally dependent on different characteristics like curvature, image gradient, and image statistical information. The existing active contour models can be classified into two categories: edge-based models [7–10] and region-based models [11–19]. These two types of models have their own advantages and disadvantages, and the choice between them in applications depends on the different characteristics of images. The edge-based model builds an edge stopping function using image edge information, which enforces the evolution of contour towards the object boundary. A balloon force term is used in the contour evolution process, which helps the contour to shrink or expand. The selection of an accurate balloon force is main problem in this method [18]. Furthermore, for the images with intense noise or weak edges, the edge stopping function based on the image gradient can hardly stop at correct boundaries.

On the other hand, a region-based model uses statistical information to construct a region stopping function, which stops the contour evolution between different regions. Compared to an edge-based model, a region-based model performs better on images with weak or blurred edges. A region-based model is not sensitive to initialization of the level set function and can recognize object's boundaries efficiently. Therefore, region-based models, especially Chan and Vese (CV) model [11], have been widely applied for image segmentation. Although, a region-based model is better than an edge-based model in some aspects but it still has limitations. The traditional region-based models [11, 13] were proposed in the context of binary images with an assumption that input image has homogenous patterns throughout whole image domain. Therefore, such models cannot segment intensity inhomogeneous objects in an image. To solve this problem Zhang et al. proposed a new region-based active contour method [12], which uses the advantages of both CV and geodesic active contour (GAC) models.

Reinitialization, a technique used for occasionally reinitializing a level set function to a signed distance function (SDF) during the evolution, has been extensively used as a numerical remedy for maintaining stable curve evolution and ensuring desirable results. From a practical viewpoint, the reinitialization process can be quite convoluted and expensive and can have subtle side effects [20]. Zhang et al. proposed the active contours with selective local or global (ACSLG) segmentation method, which uses a Gaussian kernel to regularize the level function after each iteration step. It not only regularizes the level set but also removes the need of computationally expensive reinitialization. An edge-based active contour model gives very poor results for the images with intense noise and weak edges, while on the other hand a region-based model gives no satisfactory result for the images with the intensity inhomogeneity. In this paper, a new region-based model is proposed for image segmentation and contour maps computation by incorporating the advantages of algorithms the level set evolution without reinitialization (LSEWR) [9], CV [11], and ACSLG [12] models. The proposed model uses statistical information inside and outside of the contour to construct a region-based signed pressure force (SPF) function, which controls the direction of contour evolution. In the formulated energy function this SPF function substitutes the edge indicator function in LSEWR model.

The proposed SPF function having opposite signs across the object boundary helps level set to shrink and expand. Contour shrinks if the initial contour is outside the boundary of the object and it expands if initial contour is inside the object boundary. In the proposed SPF function a mask term is used to restrict the contour movement inwards. That mask term helps to select the inner region and discard the outer region during the contour evolution process. The proposed algorithm partitions an image into two subregions and then inner part of the subregions is partitioned further into two subregions iteratively and so on until a stopping condition is fulfilled. Subregions are detected through the minimization of a new energy model restricted to a characteristics function of a subregion. If *n* is the number of iterations in order to attain final segmentation result, then *n* + 1 would be total numbers of regions from initial to final contour.

The introduced model embeds an SPF function based on a traditional region-based model [11] to target images with intensity inhomogeneity. The traditional region-based model [11] cannot properly segment image with intensity inhomogeneity because it cannot differentiate small intensity differences between two consecutive regions and cannot detect weak object boundaries. In the proposed algorithm, the proposed SPF function by using a mask term can control the contour direction and contour stopping algorithm can control the stopping point between two consecutive contours. The proposed algorithm replaced an edge indicator function in LSEWR model with an SPF function to introduce a new region-based model to trace down high intensity ambiguous regions in medical images.

The proposed algorithm contemporaneously traces high intensity or dense regions, which are tumors, masses, or salient dense tissues in medical image modalities. The resulting representation establishes an analysis of the global structure of region of interest. The contour shrinkage depends on the intensity of the region. If intensity difference between background and desired object is high then contour will evolve quickly; otherwise, the contour evolution will take time. The proposed method is good at finding high intensity and dense regions in an image. Therefore, it can properly segment salient dense regions, tumors, polyps, and blood rupture veins. Segmentation of cancer tissues in mammograms, brain tumors in brain magnetic resonance (MR) images, and ruptured blood vessels analysis in the angiography are some of the applications in which proposed method can be used. The formulated algorithm has been applied to different real images in order to demonstrate the accuracy, effectiveness, and robustness of the algorithm. Furthermore, the proposed segmentation algorithm can also be used to produce an adaptive contour map for the topographic analysis of objects in medical images.

## 2. Related Work

In [13] Mumford and Shah formulated the image segmentation problems as follows: find an optimal piecewise smooth approximation function of *u* of image *I*, which varies smoothly within each subregion *Ω*_*i*_ of the image domain *Ω*_*i*_ ⊂ *R* ^2^ and rapidly or discontinuously goes across the boundaries of *Ω*_*i*_. They proposed the following energy functional:

(1) EMSu,C=λ∫ΩI−ux2dx +v∫Ω∖C∇u2dx+μLengthC,

where |**C**| is the length of the contour *C* and *μ* and *v* ≥ 0 are fixed parameters. The unknown set *C* and the nonconvexity of the above energy functional make it difficult to be minimized. Some alternative methods have been proposed to simplify or modify the above functional, introduced as follows.

Chan and Vese [11] proposed an active contour method (ACM) based on the Mumford and Shah model [13]. Let *I* : *Ω* → *R* be an input image and let *C* be a closed curve; the energy functional is defined by

(2) ECVϕ,c1,c2=λ1∫ΩIx−c12Hεϕxdx +λ2∫ΩIx−c221−Hεϕxdx +μLengthC+vAreainC,

where *μ* ≥ 0, *v* ≥ 0, *λ*_1_, and *λ*_2_ > 0 are fixed parameters. The Euclidean length term is used to regularize the contour. *c*_1_ and *c*_2_ are two constants that approximate the image intensities inside and outside of the contour *C*, respectively. Minimizing the above energy functional by using the steepest gradient descent method [21] and representing the contour *C* with zero level set, that is, *C* = {*x* ∈ *Ω*∣*ϕ*(*x*) = 0}, we obtain the following variational formulation:

(3) $$\begin{array}{c} c_{1}=\frac{\int_{\Omega }^{}I\left(x\right)H_{\varepsilon }\left(\phi \left(x\right)\right)dx}{\int_{\Omega }^{}H_{\varepsilon }\left(\phi \left(x\right)\right)dx}, \end{array}$$

(4) ∂ϕ∂t=+μdiv∇ϕ∇ϕ−v−λ1I−c12+λ2I−c22  +μdiv⁡∇ϕ∇ϕ−vδεϕ.

The data fitting term −*λ*_1_(*I* − *c*_1_)^2^ + *λ*_2_(*I* − *c*_2_)^2^ plays a key role in curve evolution, and *λ*_1_ and *λ*_2_ govern the trade-off between the first and second term. In most cases, we set *λ*_1_ = *λ*_2_ and *v* = 0. *μ* is a scaling parameter. If it is small enough, then small objects are likely to be extracted; if it is large, big objects can be detected [11]. Obviously, in 4, *c*_1_ and *c*_2_ are related to the global properties of the image contents inside and outside the contour, respectively. However, such global image segmentation is not accurate if the image intensity inside or outside the contour is inhomogeneous.

In [12] proposed a region-based active contour method. First a new region-based signed pressure force function is proposed, which efficiently stop the contours at weak or blurred edges. Second the exterior and interior boundaries are automatically detected with the initial contour being elsewhere in the image. Third the proposed ACM with selective binary and Gaussian filtering regularized level set has the property of selective local or global segmentation. It can segment not only the desired object but also the other objects. The computational cost for the traditional reinitialization is reduced. Finally, the proposed algorithm is efficiently implemented by the simple finite difference scheme.

Let *Ω* be a bounded open subset of *R* ^2^ and let *I* : [0, *a*]×[0, *b*] → *R* ^+^ be a given image. Let *C*(*q*):[0, 1] → *R* ^2^ be a parameterized planar curve *Ω* in image domain. The GAC is formulated by minimizing the following energy functional:

(5) $$\begin{array}{c} E_{\text{GAC}}\left(C\right)=\overset{1}{\underset{0}{\int }}g\left(\left|\nabla I\left(C\left(q\right)\right)\right|\right)\left|C^{\prime }\left(q\right)\right|dq. \end{array}$$

Using calculus of variation, we get the following Euler Lagrange equation:

(6) $$\begin{array}{c} C_{t}=g\left(\left|\nabla I\right|\right)\kappa \vec{N}-\left(\nabla g\cdot \vec{N}\right)\vec{N}, \end{array}$$

where *κ* is the curvature of the contour computed across the boundary of the system and $\vec{N}$ is the normal computed inwards to the computed contour. Normally a velocity term *α* is added in order to accelerate the evolution process of the contour. Then the above equation can be rewritten as

(7) $$\begin{array}{c} C_{t}=g\left(\left|\nabla I\right|\right)\left(\kappa +\alpha \right)\vec{N}-\left(\nabla g\cdot \vec{N}\right)\vec{N}. \end{array}$$

The corresponding level set formulation will be as follows:

(8) $$\begin{array}{c} \frac{\partial \phi }{\partial t}=g\left|\nabla \phi \right|\left(\mathrm{div}\left(\frac{\nabla \phi }{\left|\nabla \phi \right|}\right)+\alpha \right)+\nabla g\cdot \nabla \phi . \end{array}$$

The SPF function defined in [22] is in the range [−1, 1]. It modulates the sign of pressure force inside and outside of the region of interest and it is used to shrink the contour when outside the object and expands it when inside the object. The mathematical formulation of the proposed SPF function is as follows:

(9) $$\begin{array}{c} \text{spf}\left(I\right)=\left\{\begin{array}{cc} \frac{I\left(x\right)-\left(c_{1}+c_{2}\right)/2}{\max\left(\left|I\left(x\right)-\left(c_{1}+c_{2}\right)/2\right|\right)}, & I\left(x\right)\neq 0\\ 0, & I\left(x\right)=0. \end{array}\right. \end{array}$$

By replacing the edge indicator function with the sign pressure force function we get the following formulation:

(10) ∂ϕ∂t=spfIx∇ϕdiv⁡∇ϕ∇ϕ+α +∇spfIx·∇ϕ.

In [12] a new regularization method is introduced for the level set regularization; therefore, curvature term can be removed.

In addition, the term ∇spf · ∇*ϕ* in above equation can also be removed, because their model utilizes the statistical information of regions, which has a larger capture range and capacity of antiedge leakage. Finally, the level set formulation of the proposed model can be written as follows:

(11) $$\begin{array}{c} \frac{\partial \phi }{\partial t}=\text{spf}\left(I\left(x\right)\right)\cdot \alpha \left|\nabla \phi \right|. \end{array}$$

In [19] a region-based active contour method is proposed, which utilizes an SPF function based on a traditional active contour method [11]. The proposed method uses both region and edge-based information in the implementation of the energy formulation. It is formulated by using edge-based model [9] as a base and by replacing the edge indicator function with a region-based SPF function, which was formulated using traditional active contour method [11].

Let *I* : *Ω* → *R* be an input image and let *C* be a closed curve; the energy functional is defined by

(12) $$\begin{array}{c} E_{g,\lambda ,v}\left(\phi \right)=\lambda L_{g}\left(\phi \right)+vA_{g}\left(\phi \right)+\mu P\left(\phi \right), \end{array}$$

where *L*_*g*_ is length term of the level set curve, *A*_*g*_ is the area term which deals with the area across the object of interest, and *P* is the penalizing term used to remove the penalizing energy during the level set curve evolution process:

(13) Eg,λ,vϕ=λ∫Ωgδϕ∇ϕdx dy +v∫ΩgH−ϕdx dy +μ∫Ω12∇ϕ−12dx dy.

By replacing the edge indicator function *g* with an SPF function based on a traditional region-based active contour method we get the following formulation:

(14) Eg,λ,vϕ=λ∫Ωgδϕ∇ϕdx dy +v∫ΩspfIH−ϕdx dy +μ∫Ω12∇ϕ−12dx dy.

By the calculus of variations [21] the steepest descent process for minimization of the energy functional *E* is the following gradient flow:

(15) ∂ϕ∂t=λδϕdiv⁡g∇ϕ∇ϕ+v spfIδϕ +μΔϕ−div⁡∇ϕ∇ϕ,

where Δ is the Laplacian operator used in the energy penalization step during the evolution of level set curve.

## 3. The Proposed Region-Based Active Contour Method for Segmentation of Region of Interest and Contour Mapping

A curve *C* in *Ω* is represented by a level set function *ϕ* : *Ω* → *R*, which is zero *ϕ* = 0 at object boundary in image *I*. Curve *C* partitions a subregion *W*_*k*_ ⊂ *Ω* into two subregions *w*, $\overset{-}{w}$ with *ϕ*, such that

(16) $$\begin{array}{c} \text{inside}\left(C\right)=w=\left\{x\in W_{k}:\phi \left(x\right)>0\right\},\\ \text{outside}\left(C\right)=\overset{-}{w}=\left\{x\in \Omega :\phi \left(x\right)<0\cup x\in \Omega \setminus W_{k}\right\}. \end{array}$$

In the proposed algorithm, the evolution of the level set starts from the initial level set function and goes on by moving the contour inwards. In order to compute the contour map of the image the initial contour is defined at the boundary of the image. To move the level set inwards, we calculate the inner subregion of zero level curve using *ϕ* > 0.

**Figure 1:**
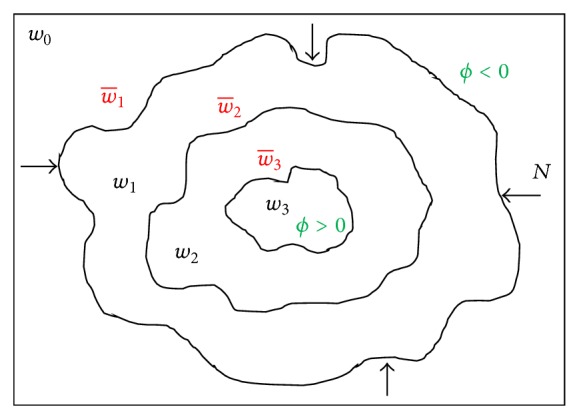
Calculated isocontour map from a given image *w*_0_.

Figure 1 shows an isocontour map image in which the inner regions *w*_1_, *w*_2_, and *w*_3_ are calculated using *ϕ* > 0, whereas the outer regions are calculated by subtracting the energy component of the current calculated inner subregion from the previous calculated inner region, as shown in the equations below:

(17) w0=w1+w−1⟹w−1=w0−w1,w−2=w1−w2,w−3=w2−w3.

In general, the outer subregion can be calculated as

(18) $$\begin{array}{c} {\overset{-}{w}}_{k}=w_{k-1}-w_{k}. \end{array}$$

In image segmentation, active contours are dynamic curves that move toward the object boundaries. To achieve this goal, we explicitly define an external energy that can move the zero level set towards the object boundaries. We define energy functional for function *ϕ* as follows:

(19) $$\begin{array}{c} E_{\text{spf}}\left(\phi \right)=\lambda L_{\text{spf}}\left(\phi \right)+vA_{\text{spf}}\left(\phi \right), \end{array}$$

where *λ* > 0 and *v* are constants, and the terms *L*_spf_(*ϕ*) and *A*_spf_(*ϕ*) are defined as below:

(20) $$\begin{array}{c} L_{\text{spf}}\left(\phi \right)=\int_{\Omega }\text{spf}\left(I\right)\delta_{\varepsilon }\left(\phi \right)\left|\nabla \phi \right|dx, \end{array}$$

(21) $$\begin{array}{c} A_{\text{spf}}\left(\phi \right)=\int_{\Omega }\text{spf}\left(I\right)H_{\varepsilon }\left(-\phi \right)dx, \end{array}$$

respectively. Here, spf(*I*) is the proposed SPF function defined in 29, while *H*_*ε*_ is the Heaviside function and *δ*_*ε*_ = *H*_*ε*_′ is the univariate Dirac delta function given in 30 and 31, respectively. The zero level curve *C* is driven into a smooth curve from a complicated curve to minimize the function *L*_spf_(*ϕ*). The small energy of *A*_spf_(*ϕ*) accelerates the evolution. The SDF satisfies the desirable property |∇*I*| = 1. The energy functional *E*_spf_(*ϕ*) drives the zero level set toward the object boundaries. The coefficient *v* of *A*_spf_(*ϕ*) in 21 can be positive or negative, depending on the relative position of the initial contour to the object of interest. For example, if the initial contours are placed outside the object, the coefficient *v* in the weighted area term should take a positive value, so that the contour can shrink faster. If the initial contours are placed inside the object, the coefficient *v* should take a negative value to speed up the expansion of the contours.

We define a new region-based energy functional based on CV [11] as shown in 2 with an additional mask term *M* ^*k*^ to restrict the contour evolution inwards. We proposed *E*_proposed_ in order to formulate the SPF function that is used in 20 and 21. Let *I* : *Ω* → *R* be an input image and let *C* be a closed curve; the energy functional *E*_proposed_ is defined by

(22) Eproposed=∫ΩIx−c12HεϕxMkxdx +∫ΩIx−c221−HεϕxMkxdx.

Keeping *ϕ* fixed and minimizing the energy *E*_proposed_ with respect to *c*_1_ and *c*_2_, it is easy to express these constant functions of *ϕ*; we get *c*_1_ and *c*_2_ of regions *w* and $\overset{-}{w}$, respectively, as follows:

(23) $$\begin{array}{c} c_{1}\left(\phi \right)=\frac{\int_{\Omega }^{}I\left(x\right)H_{\varepsilon }\left(\phi \left(x\right)\right)M^{k}\left(x\right)dx}{\int_{\Omega }^{}H_{\varepsilon }\left(\phi \left(x\right)\right)M^{k}\left(x\right)dx}, \end{array}$$

(24) $$\begin{array}{c} c_{2}\left(\phi \right)=\frac{\int_{\Omega }^{}I\left(x\right)\left(1-H_{\varepsilon }\left(\phi \left(x\right)\right)\right)M^{k}\left(x\right)dx}{\int_{\Omega }^{}\left(1-H_{\varepsilon }\left(\phi \left(x\right)\right)\right)M^{k}\left(x\right)dx}. \end{array}$$

Here, *M* ^*k*^ is the characteristic function of subregion *W*_*k*_, defined as

(25) $$\begin{array}{c} M^{k}\left(x\right)=\phi >0,\\ M^{0}:\Omega ⟶-1. \end{array}$$

By the calculus of variations [21], the Gateaux derivative (first variation) of the functional *E*_spf_(*ϕ*) in 19 can be written as

(26) $$\begin{array}{c} Q\left(\phi \right)=\left(\lambda \cdot \mathrm{div}\left(\text{spf}\left(I\right)\cdot \frac{\nabla \phi }{\left|\nabla \phi \right|}\right)+v\cdot \text{spf}\left(I\right)\right)\delta_{\varepsilon }\left(\phi \right). \end{array}$$

The function *ϕ* that minimizes this functional satisfies the Euler Lagrange equation ∂*E*_spf_/∂*ϕ* = 0. The first term corresponds to the *L*_spf_(*ϕ*) (weighted length term), which deals with curvature of the object boundary based on edge information, whereas the second term *A*_spf_(*ϕ*) (weighted area term) is used to compute the area of regions of interest in image inside of the initial contour. If the SPF value is positive, then contour moves toward the high intensity region and vice versa.

A classical iterative process for minimizing the function *E*_spf_(*ϕ*) is the gradient flow with artificial time *t* given as

(27) $$\begin{array}{c} \phi_{\left(t=0\right)}=\phi_{0},\;\;\frac{\partial \phi }{\partial t}=Q\left(\phi \right). \end{array}$$

After evolving the level set using 26 and 27 we regularize it by using *ϕ* ^*n*^ = *G*_*σ*_∗*ϕ* ^*n*^. It not only regularizes the level set function but also eliminates the need of reinitialization, which is computationally very expensive. Regularization using Gaussian kernel has better smoothing results and no energy leakage as compared to the area smoothing and penalization terms used by Li et al. [9].

An SPF function is a mathematical expression whose value is in the range [−1, 1] inside and outside of region of interest. Numerous ways have been devised to formulate an SPF function [12, 23, 24] some of them incorporate global intensity means and some use local intensity means in its construction. In this paper we propose a new SPF function, which uses global fitted image restricted with a mask term to enforce level set evolution inwards. If mask term is set to 1 then it modulates the signs of the pressure forces inside and outside the region of interest so that the contour shrinks when outside the object and expands when inside the object. A two-phase global fitted image model is for a level set function defined as follows:

(28) $$\begin{array}{c} I_{\text{GFI}}=c_{1}H_{\varepsilon }\left(\phi \right)+c_{2}\left(1-H_{\varepsilon }\left(\phi \right)\right). \end{array}$$

Using the global fitted model defined above we constructed the SPF function as follows:

(29) $$\begin{array}{c} \text{spf}\left(I\right)=\left\{\begin{array}{cc} \frac{\left(I\left(x\right)-I_{\text{GFI}}\right)M^{k}}{\max\left(\left|I\left(x\right)-I_{\text{GFI}}\right|\right)} & I\left(x\right)\neq 0,\\ 0 & I\left(x\right)=0. \end{array}\right. \end{array}$$

**Figure 2:**
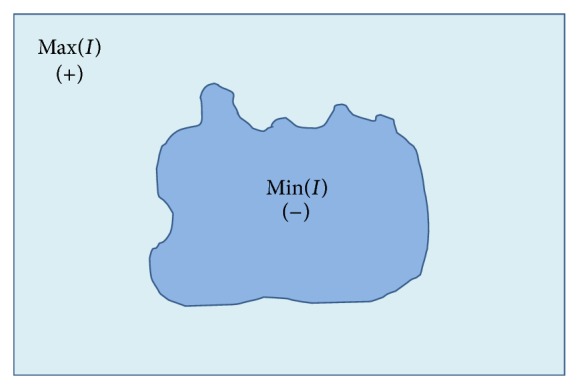
The signs of the SPF function inside and outside the object are opposite one another.

The terms *c*_1_(*ϕ*),  *c*_2_(*ϕ*), and *M* ^*k*^ are defined in 23, 24, and 25, respectively. The signs of SPF function are shown in Figure 2, which is negative for the inside region and positive for the outside region, considering an inside region with higher intensity than the outer one. The mask term *M* ^*k*^ from 25 is to restrict the evolution of contour inside of the object boundary.

In the proposed work, the Dirac function *δ*_*ε*_(*z*) and Heaviside function *H*_*ε*_(*z*) used in 23, 24, and 26 are the smoothed versions of the Dirac function and Heaviside function over the entire region. The approximations *H*_*ε*_(*z*) and *δ*_*ε*_(*z*) as proposed in [11] are

(30) $$\begin{array}{c} H_{\varepsilon }\left(z\right)=\frac{1}{2}\left(1+\frac{2}{\pi }\arctan\left(\frac{z}{\varepsilon }\right)\right), \end{array}$$

(31) $$\begin{array}{c} \delta_{\varepsilon }\left(z\right)=\frac{\varepsilon }{\pi \left(z^{2}+\varepsilon^{2}\right)}. \end{array}$$

We use the regularized Dirac *δ*_*ε*_(*z*) and Heaviside *H*_*ε*_(*z*) with *ε* = 1.5 for all the experiments in this paper, and the curvature term is computed using the central difference method.

In outmoded level set methods, it is essential to initialize the level set function *ϕ* as a signed distance function (SDF) *ϕ*_0_. If the initial level set function is expressively different from the SDF, then the reinitialization schemes are unable to reinitialize the function to the SDF. In our formulation, not only is the reinitialization procedure completely eliminated, but the level set function *ϕ* also no longer needs to be initialized as SDF. The initial level set function *ϕ*_0_ is defined as

(32) $$\begin{array}{c} \phi \left(x,t=0\right)=\left\{\begin{array}{cc} -\rho & x\in \Omega_{0}-\partial \Omega_{0},\\ 0 & x\in \partial \Omega_{0},\\ \rho & x\in \Omega -\Omega_{0}, \end{array}\right. \end{array}$$

where *ρ* > 0 is a constant and we use *ϕ*_0_ with *ρ* = 2.

In order to stop the contour at certain point a stopping algorithm is used, which checks the similarity of pixels between two consecutive contours using stopping value. If

(33) $$\begin{array}{c} \overset{\text{row}}{\underset{i=0}{\sum }}\;\overset{\text{col}}{\underset{j=0}{\sum }}M_{i,j}^{k}<\left(\frac{\text{stopping}\;\text{value}}{100}\right)\overset{\text{row}}{\underset{i=0}{\sum }}\;\overset{\text{col}}{\underset{j=0}{\sum }}\text{old}M_{i,j}^{k} \end{array}$$

then contour will stop moving any further, where old *M* ^*k*^ is the mask term of the last computed contour, *M* ^*k*^ is the mask term of the current computed contour, row is maximum number of rows, and col is maximum number of columns of the input image. The stopping value is always 98 < stopping  value < 100 and computed by calculating the mean intensity value from the initial contour with the scale always in between 98 and 100. A threshold value *T* is used to remove small values while calculating stopping value. In the experiments related to mammograms we selected *T* = 25.

Finally, the principle steps of the algorithm can be summarized as follows.

- Initialize *ϕ* by −*ϕ*_0_ and *M* ^*k*^ by −*M* ^0^, using 32 and 25, respectively, at *k* = 0.
- Compute *c*_1_(*ϕ*) and *c*_2_(*ϕ*) from 23 and 24, respectively.
- Calculate spf(*I*) using 29.
- Solve the partial differential equation (PDE) in *ϕ* from 26 and 27, to obtain *ϕ* ^*k*^.
- Calculate *M* ^*k*^ using the previously calculated *ϕ* ^*k*^ value (in next iteration, which is *ϕ* ^*k*−1^).
- Regularize the level set function by a Gaussian kernel; that is, *ϕ* ^*k*^ = *G*_*σ*_∗*ϕ* ^*k*^, where *σ* is standard deviation.
- Check whether solution is stationary using stopping algorithm discussed above. If not, go to step (b), *k* = *k* + 1, and repeat.

If one of the subregions *w*, $\overset{-}{w}$ is empty, then the formulation degenerates and the algorithm automatically terminates.

**Figure 3:**
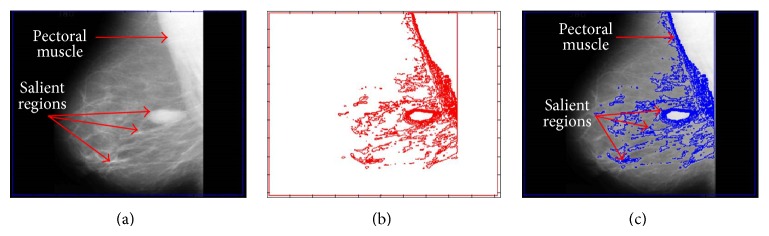
Isocontour map with step size of 50 iterations. (a) Initial contour nearby the boundary of image. (b) Isocontour map for 400 iterations. (c) Isocontour map on given mammogram image for 400 iterations.

Although the main objective of the proposed method is to extract regions of interest from medical images it can also provide step by step contour information to build a contour map for the topographic analysis of a medical image. Using the contour map we can analyze the structure of a given image in more detail and extract the meaningful information more easily.  Figure 3 shows how contour map helps in analyzing and pinpointing the salient region in a mammogram image with salient regions.

Figure 3(a) shows the original image with the initial contour.  Figure 3(b) shows the contour map of 400 iterations with a step size of 50 iterations on a masked image and Figure 3(c) shows the computed contour map on the given image. In Figure 3(c) we can identify the salient regions with the area that contains thick contours. Or simply we can say that the salient regions are the regions in the contour map image where consecutive contour lines are dense or very close to each other. In Figure 3(a) salient regions are identified with arrows, where the top arrow identifies the pectoral muscle, which we need to ignore. Figure 3(c) verifies the dense contour lines for salient regions.

**Figure 4:**
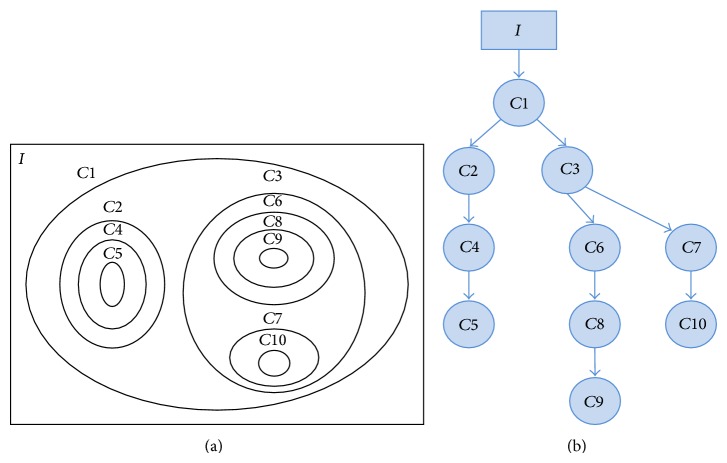
Schematic illustration of the inclusion tree. (a) Isocontour map containing branching contour and base contours. (b) Corresponding inclusion tree.

The computed contour map can further be used to form a contour tree which is a way to analyze topographic data in more depth. A contour tree is a graph that tracks contours of the level set as they split, join, appear, and disappear [25]. This provides a hierarchical representation of the enclosure relationship between isocontours. In the contour tree, each node represents a contour at a parent node spatially that encloses the contour at its descendent nodes. A node is classified as branching or nonbranching depending on its degree. A node with more than one intermediate descendent node is called a branching node, and a nonbranching node has only one or zero immediate descendent nodes. The contours at the branching nodes in the inclusion tree are called branching contours and the contours at their intermediate descendants are called base contours. In particular, contours at the terminal nodes that have no further child due to fulfilled stopping condition are called terminal contours [2]. The concept of inclusion tree is introduced here because one of the applications of the proposed algorithm is to create contour tree from the developed contour map. A schematic illustration of the isocontour map is shown in Figure 4(a), while Figure 4(b) shows the inclusion tree of the isocontour map; here, contours *C*1 and *C*3 are branching contours, contours *C*2, *C*6, and *C*7 are base contours, and contours *C*5, *C*9, and *C*10 are terminal contours.

We can measure the saliency of contour using minimum nesting depth from inclusion tree. The nesting depth for a contour is given by the number of contours from the innermost contour to the contour within the nesting structure. The base contours with higher minimum nesting depth correspond to the boundaries of distinctive regions with abrupt intensity changes.

## 4. Results

The proposed technique is applied to the mammogram images from the mini-MIAS database [26]. The range of intensities in all images is represented from 0 to 255, while the size in pixels (length × width) of the images is 1024 × 1024. The used experiment environment is Windows 7, Quad Core CPU 2.4 GHz, and 8 GB RAM. In the proposed method the following parameters are used: *K* = 5, *σ* = 1, *λ* = 1.0, *v* = 15, and Δ*t* = 1, where Δ*t* is a time step in the numerical implementation of 27. *K* is the width of the Gaussian kernel and *σ* is the standard deviation, which uses all points around the center point to make the level set function smooth.

**Figure 5:**
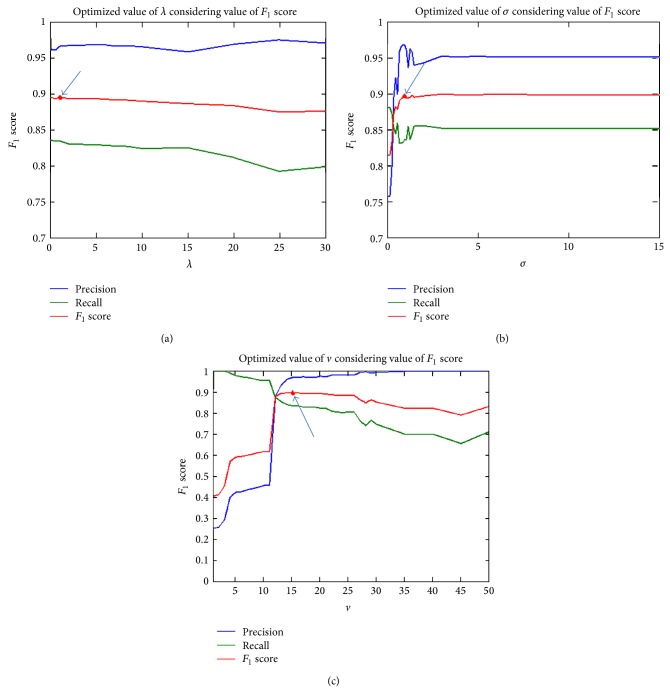
Finding optimal parameters for the proposed method using *F*_1_ score analysis. (a) *F*_1_ score at different value of *λ* (keeping *σ* and *v* constant), (b) *F*_1_ score at different value of *σ* (keeping *λ* and *v* constant), and (c) *F*_1_ score at different value of *v* (keeping *λ* and *σ* constant).

In the proposed method parameters are selected manually. In order to achieve optimized values a small analytical experiment is conducted using a breast image (mdb005) from the mini-MIAS database. In the proposed method *λ*, *σ* and *v* are the main values, which affect the evolution of contour and its accuracy. In order to find best values of *λ*, *σ*, and *v*, *F*_1_ score (*F* measure) is computed at different values of *λ*, *σ*, and *v*. *F*_1_ score is a measure to compute accuracy of a method, its value is close to 1 when method has high accuracy, and its value is close to 0 when method has low accuracy. The mathematical expression to compute *F*_1_ score is as follows:

(34) $$\begin{array}{c} F_{1}=2\cdot \frac{\text{Precision}\cdot \text{Recall}}{\text{Precision}+\text{Recall}}, \end{array}$$

where precision is the number of correct positive results divided by the number of all positive results and recall is the number of correct positive results divided by the number of positive results that should have been returned. The mathematical formulation of precision and recall is given in Section 5. Here, *F*_1_ score is computed for all three parameters (*λ*, *σ*, and *v*) one by one by keeping two parameters constant and varying one parameter on a certain scale to see on which parameter *F*_1_ score is close to 1.  Figure 5 shows precision, recall, and *F*_1_ score of proposed method (applied on mdb005 from mini-MIAS database) at different values of *λ*, *σ*, and *v*. Precision and recall should also be close to 1 for high accuracy. *F*_1_ score is harmonic mean of precision and recall; therefore, if *F*_1_ score is highest at some point then collective weight of precision and recall at that point is also high. Here, in Figure 5 we will only study *F*_1_ score because its value is representing both precision and recall.

Figure 5(a) shows *F*_1_ score of the proposed method at different values of *λ* (keeping *σ* and *v* constant). It shows that at *λ* = 1  *F*_1_ score has maximum value, which means it provides best result at *λ* = 1. From 0 ≤ *λ* ≤ 6 contour is stable but for all values at *λ* > 6 contour evolution is unstable in first iterations because of oversaturation of energy in length term.  Figure 5(b) shows *F*_1_ score has maximum at *σ* = 1 and *σ* = 1.4. In all values at *σ* > 0.8  *F*_1_ score is almost same. We choose *σ* = 1 because at small *σ* algorithm has less time complexity. If we increase *σ* window size of the smoothing kernel will also be increased, which will also add up the computations and increase the time complexity of the algorithm.  Figure 5(c) shows that *F*_1_ score has maximum value at *v* = 15. From the *F*_1_ score analysis we found that at *λ* = 1, *σ* = 1, and *v* = 15 the proposed method gives high accuracy; therefore, we set the parameters to these values.

**Figure 6:**
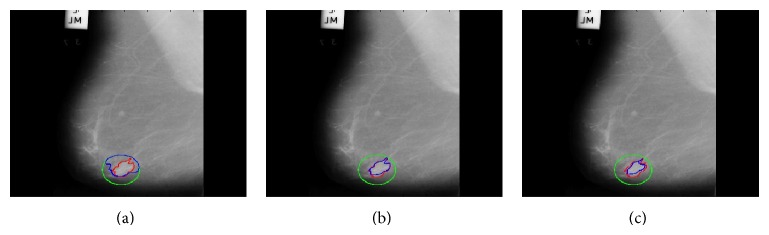
Segmentation results at different *v*. (a) At *v* = 5 with small step size, (b) at *v* = 15 with medium step size, and (c) at *v* = 30 with big step size.

Here, *v* is the most import parameter, which controls the scaling of the area term in the proposed algorithm. By changing the value of *v*, the step size between two consecutive contours can be controlled, which can affect the segmentation accuracy of the region of interest.  Figure 6 shows segmentation results using different values of *v*. It shows that at *v* = 5 when step size is small contour stop evolving earlier than expected, while at *v* = 30 when step size is big contour stopped later than expected and at *v* = 15 more accurate segmentation results are achieved.

**Figure 7:**
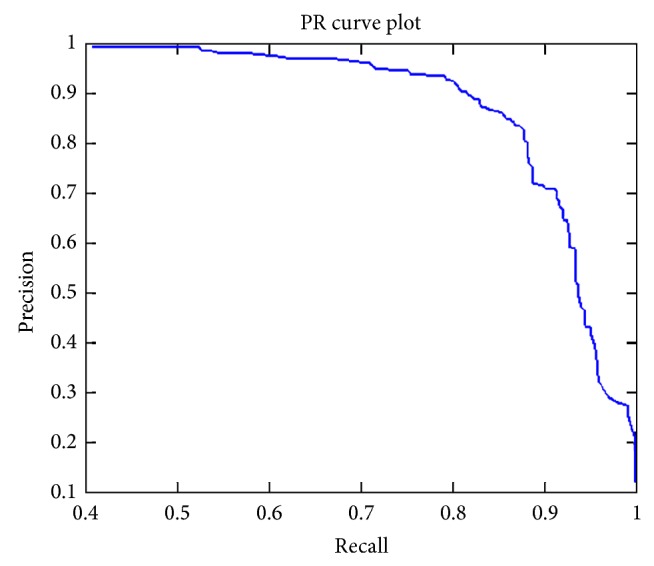
PR curve using precision and recall from segmentation results of 116 images with tumor tissues from mini-MIAS database.

**Figure 8:**
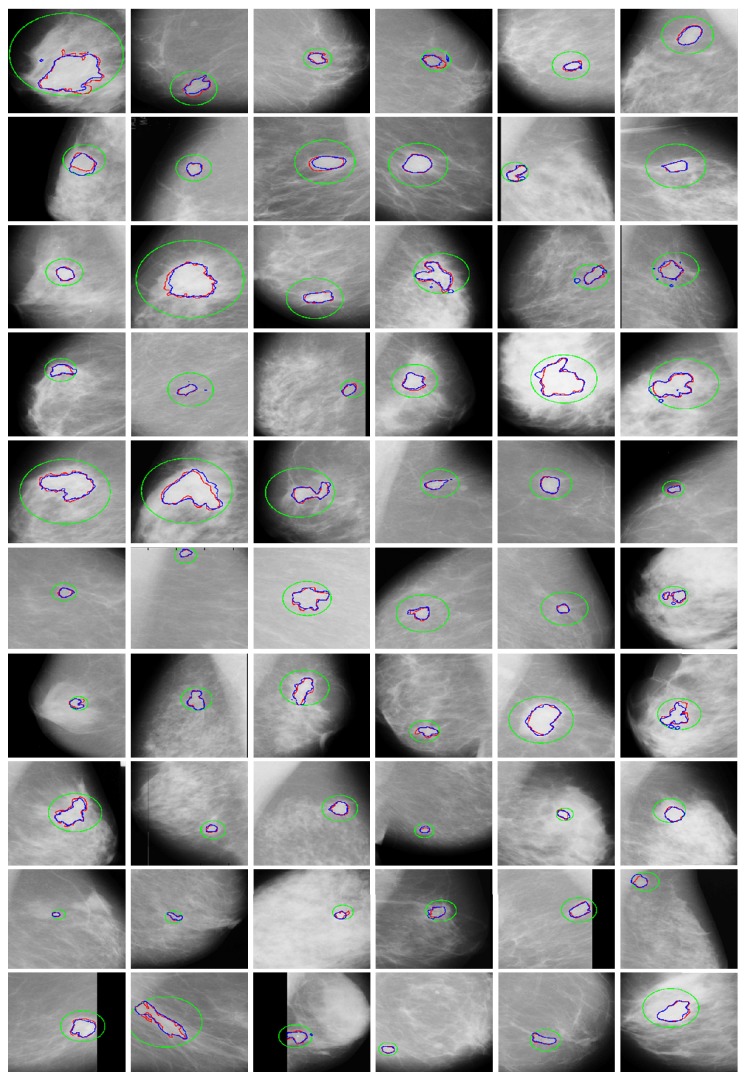
Examples of successful tumor tissue segmentation from mini-MIAS database. The initial contour is shown in green, the ground truth contour is shown in red, and the computed segmentation of region of interest is shown in blue.

The proposed method is tested on 116 images with tumor tissues out of 315 total images in the database. No ground truths of tumor tissues are pregiven in the mini-MIAS database; only the information regarding location and diameter is mentioned. In the first step of our result evaluation we drew ground truth by ourselves with hand based marker using the location and diameter information given in database. Location of region of interest is kept in mind during initialization of initial contour. It is initialized outside the ground truth region (surrounding region of interest) and not far away in order to increase the accuracy. The proposed algorithm segmented all 116 images but some with accuracy problems.  Figure 7 shows the PR curve computed using precision and recall from segmentation results of all 116 images. Visually, it shows overall accuracy of over 80%. In the computed segmentation results from 116 mammogram images, 26.72% results have precision of 0.90 or more, 28.45% results have precision of 0.80 or more, 5.17% results have precision of 0.70 or more, 12.07% results have precision of 0.60 or more, 5.17% results have precision 0.50 or more, and 21.41% result have precision less than 0.49, while among 116 of total segmented mammogram images 43.10% results have recall of 0.90 or more, 41.38% have recall of 0.80 or more, 5.17% results have recall of 0.70 or more, 5.17% results have recall of 0.60 or more, 4.27% results have recall of 0.50 or more, and 0.86% have recall less than 0.49. Moreover, maximum achieved precision and recall from segmentation results are 0.99 and 1, respectively. While minimum achieved precision and recall are 0.12 and 0.41, respectively. The maximum scale for precision and recall values is 1.  Figure 8 shows some of the well segmented tumor tissue results from the mini-MIAS database. In this figure initial contour is shown in green, ground truth contour is shown in red, and computed contour is shown in red. Note that the initial contour is always taken outside the region of interest because in the proposed algorithm contour always moves inwards discarding the outer contour.

**Figure 9:**
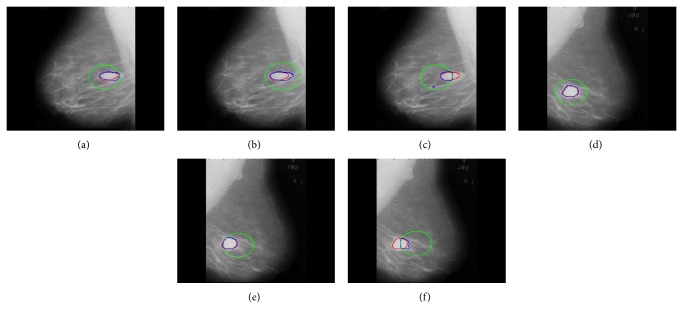
Effect of position of initial contour on segmentation of regions of interest.

Accuracy of segmentation results is not affected much if the center position of initial contour is changed but keep in mind that while changing its position it still must surround the region of interest completely.  Figure 9 shows that the position of initial contour will not affect the accuracy of segmentation results if it contains the area of region of interest within it. But the segmentation accuracy will be affected if initial contour is out of region of interest or if it is partially surrounding the region of interest. The proposed method partitions an image using 2^*n*^ region method and discards all outer regions during the contour evolution process. If the initial contour surrounds partial region of interest and remaining part appears outside of it then the region of interest which lies outside the initial contour is considered useless information by the proposed algorithm and is discarded. Therefore, the proposed algorithm's accuracy is affected when initial contour does not surround whole region of interest. In Figures 9(a), 9(b), 9(d), and 9(e) the segmentation result is not affected much though the position of initial contour is changed because changed initial contour surrounded whole region of interest, whereas in Figures 9(c) and 9(f) segmented result is affected evidently because initial contour contained partial region of interest.

## 5. Discussion

**Figure 10:**
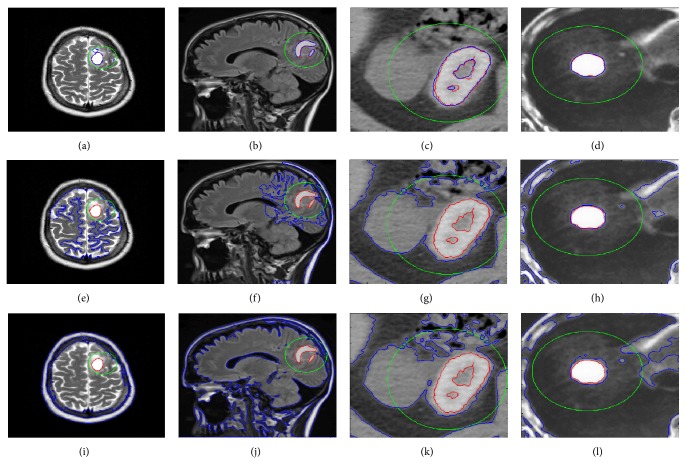
The visual results comparison between segmentation results of the proposed Zhang et al. and Jiang et al. algorithms. (a)–(d) shows initial contour in green, final segmentation results of four different images using proposed method in blue and ground truth in red. (e)–(h) shows initial contour in green, final segmentation results of four different images using Zhang et al. method in blue and ground truth in red. (i)–(l) shows initial contour in green, final segmentation results of four different images using Jiang et al. method in blue and ground truth in red.

In order to explain the advantages of the proposed method, we applied image segmentation test on four medical images shown in Figure 10. We compared the results of the proposed segmentation technique with Zhang et al. [12] and Jiang et al. [19] techniques in terms of computation time and accuracy of desired results. The parameters used for this comparison for Zhang et al. method are *μ* = 25, *ρ* = 1, *ε* = 1.5, *σ* = 1, *K* = 5, and Δ*t* = 1, while the parameters used for Jiang et al. method are *μ* = 0.04, *λ* = 3, *v* = 1, *ρ* = 2, *ε* = 1.5, and *τ* = 5. And the parameters used for the proposed method are same as mentioned in the Section 4.

Figure 10 shows a visual based comparison between the segmentation results of the proposed algorithm with Zhang et al. and Jiang et al. algorithms. Figures 10(a)–10(d) show the segmentation results produced by the proposed algorithm, Figures 10(e)–10(h) show the segmentation results produced by Zhang et al. algorithm, and Figures 10(i)–10(l) show the segmentation result computed by Jiang et al. algorithm. In Figure 10, four images from different image modalities are used with tumor tissues as regions of interests. We can see from the visual results the proposed algorithm provided well segmentation results of the desired region of interests in comparison with their respective ground truth, while the Zhang et el. and Jiang et al. methods which are in particular general segmentation method could not deliver well segmentation results of regions of interest as compared to their respective ground truths.  Table 1 shows a quantitative analysis based on the computed segmentation results from Figure 10. First we computed false positive (fp), true positive (tp), false negative (fn), and true negative (tn) from segmented object and its ground truth analysis. In order to compare the accuracy of the segmentation we used the following statistical relationships:

(35) $$\begin{array}{c} \text{Precision}=\frac{\text{tp}}{\text{tp}+\text{fp}},\;\;\text{Recall}=\frac{\text{tp}}{\text{tp}+\text{fn}},\\ \text{True}\;\text{Negative}\;\text{Rate}=\frac{\text{tn}}{\text{tn}+\text{fp}},\\ \text{Accuracy}=\frac{\text{tp}+\text{tn}}{\text{tp}+\text{tn}+\text{fp}+\text{fn}}, \end{array}$$

The term precision, which shows how much of the segmented region is same as ground truth, in terms of true positive, is the most important among all. The term recall shows whether the information that we obtain contains the information what we need, without considering false positives. The negative rate term tells us how much of true negative region is correctly ignored during segmentation. Accuracy term shows the accuracy of the segmentation result over whole image domain. The proposed method provides more precision rate, high true negative rate, high accuracy, and slightly less recall as compared to other methods.  Table 1 shows the proposed method provided better precision and almost same recall as compared to Zhang et al. and Jiang et al. methods.

## 6. Conclusion

In this paper a region-based image segmentation technique is proposed in order to detect and segment regions of interest in medical image methodologies. A region of interest corresponds to distinctive areas that may include tumor, blood rupture area, breast boundary, masses, and other dense tissue regions.

The proposed segmentation algorithm is designed to partition an image into an arbitrary number of subregions. It starts by dividing the input image into two subregions and then one of the subregions is further divided into two subregions and so on until the stopping condition is fulfilled. It is implemented with the level set method proposed by Li et al., replacing the edge indicator function with a new region-based SPF function developed from the Chan-Vese (CV) energy functional model, which efficiently stops the contours at weak or blurred edges. A Gaussian kernel is used which not only regularizes the level set function but also eliminates the need of computationally expensive reinitialization.

Contour maps can also be produced using the proposed algorithm and with the help of contour maps we can built contour trees. These tree structures can help us to analyze the topological and geometrical relationships between different contours. With the help of contour tree we can analyze the regions of interest, and we can compute the saliency of salient regions using minimum nesting depth.

The experimental results show that the segmentation of the regions of interest in an image is highly dependent on the intensity, brightness and contrast of the objects and background. The greater the intensity difference between the salient object and the rest of the image is, the more accurate the segmentation results will be because intensity based active contour moves relatively slow on high intensity regions as compared to the low intensity regions.

## Figures and Tables

**Table 1:** Quantitative analysis based on Figure 10.

Method	Figure	Precision	Recall	True negative rate	Accuracy	CPU time (s)	Number of iterations
Proposed method	Brain tumor 1	0.8536	0.9799	0.9991	0.9990	28.2500	42
	Brain tumor 2	0.9511	0.9326	0.9996	0.9991	32.8125	36
	Lung cancer	0.9113	0.9777	0.9901	0.9889	17.8750	39
	Liver cancer	0.9579	0.9979	0.9986	0.9986	13.3906	31

Zhang et al.	Brain tumor 1	0.0417	1	0.8773	0.8779	16.7188	100
	Brain tumor 2	0.0635	1	0.8778	0.8788	22.7656	100
	Lung cancer	0.1683	1	0.4866	0.5350	13.7813	100
	Liver cancer	0.2494	1	0.9047	0.9076	12.4688	100

Jiang et al.	Brain tumor 1	0.0183	1	0.7128	0.7143	50.9844	100
	Brain tumor 2	0.0172	1	0.5270	0.5309	68.2344	100
	Lung cancer	0.1758	1	0.5130	0.5588	32.3700	100
	Liver cancer	0.1473	1	0.8166	0.8223	30.7031	100
